# Prognostic Role of Adaptive Immune Microenvironment in Patients with High-Risk Myelodysplastic Syndromes Treated with 5-Azacytidine

**DOI:** 10.3390/cancers17071104

**Published:** 2025-03-25

**Authors:** Zoi Tsakiraki, Aris Spathis, Anthi Bouchla, Abraham Pouliakis, Pinelopi Vryttia, Ioannis G. Panayiotides, Vasiliki Pappa, Sotiris G. Papageorgiou, Periklis G. Foukas

**Affiliations:** 1 2nd Department of Pathology, National and Kapodistrian University of Athens, Attikon University Hospital, 12462 Athens, Greece; zoi_tsa@hotmail.com (Z.T.); aspathis@med.uoa.gr (A.S.); apouliak@med.uoa.gr (A.P.); ioagpan@med.uoa.gr (I.G.P.); 2 Hematology Unit, 2nd Department of Internal Medicine, National and Kapodistrian University of Athens, Attikon University Hospital, 12462 Athens, Greece; ampouchla@med.uoa.gr (A.B.); pinelvr@med.uoa.gr (P.V.); vaspappa@med.uoa.gr (V.P.); sotpapage@med.uoa.gr (S.G.P.)

**Keywords:** immunoenvironment, myelodysplastic syndromes, 5-Azacitidine, immunohistochemistry, digital pathology

## Abstract

Background/Objectives: There are limited data regarding immunohistochemical profiling of immune cells in bone marrow trephine biopsies of patients with high-risk myelodysplastic syndromes (HR-MDS). Methods: We sought to objectively quantify, with the use of digital pathology, the density (cells/mm2) of the prominent adaptive immunity cell populations in sixty-four (64) bone marrow trephine biopsies of HR-MDS patients receiving 5-Azacytidine. We focused on CD3(+) T cells, CD8(+) cytotoxic T cells (Tc), helper T cells (Th), Foxp3(+) regulatory T cells (Tregs), CD20(+) B-cells and CD138(+) plasma cells and evaluated the presence and the number of lymphoid aggregates. A control group of twenty “non-MDS” patients was included in the study. Results: We identified a significant decrease in adaptive immune cell densities in the HR-MDS patients compared to the non-MDS controls. Increased T and Th cell densities correlated with the response to 5-Azacytidine (5-AZA) treatment. Higher T, Tc, Th and plasma cells densities and low B, Tregs and Tregs/T cells ratios correlated with increased overall survival. Reduced Tregs, Tregs/T cells, Tregs/Tc and plasma cells showed improved leukemia-free survival. A modified IPSS-R (IPSS-R-I), combining the initial IPSS-R with the immune populations’ parameters, improved overall survival and showed a double-fold increase in Cox calculated hazard ratios. Conclusions: Immunohistochemical bone marrow immune profiling represents a powerful and easily useable tool for investigating the possible role of bone marrow immune microenvironment in the pathogenesis and progression of MDS, but also its association with the response to 5-AZA treatment and clinical outcomes.

## Simple Summary

Even though immunohistochemistry is routinely used in pathology, there are limited data regarding immune profiling of trephine biopsies from patients with high-risk myelodysplastic syndromes (HR-MDS). We used digital pathology to evaluate the cell densities of adaptive immune cells in trephine biopsies from sixty-four (64) HR-MDS patients with no prior treatment and aimed to correlate these findings with response and overall survival after 5-azacitidine treatment. Increased T regulatory and B cells had adverse effects on overall survival while increased T and T helper cells were beneficial for survival. Similar results were obtained for the response to 5-azacitidine treatment. Our findings support the inclusion of immune cell densities in a modified prognostic score based on the Revised International Prognostic Scoring System (IPSS-R).

## 1. Introduction

Several studies over the last few years have demonstrated the importance of the immune microenvironment and have highlighted the complexity of immune pathophysiology’s role in the pathogenesis of myelodysplastic syndromes (MDS) [1,2,3]. Dysfunction of both innate and adaptive immunity is evident and variable factors, such as chronic inflammation, inflammaging, escape of immune surveillance, immune checkpoints and production of cytokines, among others, have been reported to play an important role in inducing hematopoietic stem cells’ (HSC) dysfunction by favoring mutagenesis and eventually MDS development [4]. Lower-risk MDS (LR-MDS) are characterized by a proinflammatory immune response with increased populations of effector T cells, such as CD4+ cells and cytotoxic T cells (Tc) [5,6]; on the other hand, high-risk (HR) MDS show a suppressive immune profile that includes the expansion of immunosuppressive cells like Tregs and myeloid-derived suppressor cells (MDSCs) [7].

5-Azacitidine (5-AZA) is the front-line treatment for HR-MDS patients (intermediate-2 or high risk, according to the International Prognostic Scoring System, IPSS) as well as the backbone of treatment for patients with acute myeloid leukemia (AML) who are ineligible for intensive chemotherapy. Treatment with 5-AZA prolongs the time until transformation into AML and the overall survival (OS) of HR-MDS patients. However, the predictive value of immune microenvironment defects in terms of disease progression and outcome after treatment with 5-AZA has not been adequately studied.

Here, we used a micro-anatomical approach to investigate the cellular composition and possible structural niches of the elements of adaptive immunity in the bone marrow microenvironment of HR-MDS. For this, we used trephine bone marrow (BM) biopsies, immunohistochemistry (IHC) and digital pathology to quantify the densities of immune cell populations, as well as the presence and the number of lymphoid aggregates and their spatial distribution. In addition, we sought to identify potential predictive immune populations that correlate to 5-AZA response and the outcome of the disease.

## 2. Materials and Methods

### 2.1. Study Population

The study encompassed sixty-four (64) trephine BM biopsies from consecutive HR-MDS patients eligible for 5-AZA administration (41 male and 23 female) with a mean age of 73 years (range, 46–86 years). Twenty (20) “non-MDS” patients (10 male and 10 female), mean age 67 (range 43–90 years old) who were diagnosed with lymphoproliferative diseases (the majority of them were high-grade B cell lymphomas) without BM invasion or prior treatment were also recruited in this study, serving as a control group. All patients provided written consent and the study was approved by the ethics committee of the “Attikon” University Hospital.

Analysis was performed on BM biopsies acquired prior to treatment initiation with 5-AZA (subcutaneously 75 mg/m^2^/day for 5-2-2 days every 28 days). Clinical parameters were recorded when available (Table 1). Overall survival (OS) and leukemia-free survival (LFS) were measured after initiation of 5-AZA therapy. The clinical response for those patients was evaluated during a period of 6 to 12 months, as it takes more than 3 cycles of therapy to achieve a clinical response to hypomethylating agents [8,9].

### 2.2. Immunohistochemistry (IHC)

IHC was performed on 3 μm deparaffinized sections. Antibodies against CD3 (DAKO, Agilent, Santa Clara, CA, USA, rabbit polyclonal, RTU), CD20 (DAKO, L26, 1/100), CD138 (DAKO, mi15, 1/50), CD8 (DAKO, c8/144b, 1/50) and FOXP3 (Zytomed, Berlin, Germany, sp97, 1/100) were used in order to interrogate the density of total T lymphocytes, B lymphocytes, plasma cells and cytotoxic and regulatory T cells, respectively. Deparaffinization, rehydration and antigen retrieval were performed using high pH (Envision Flex, DAKO). Immunostaining was performed in the DAKO Link-48 autostainer using a commercially available kit (Envision Flex DAB, Dako).

### 2.3. Digital Imaging and Cell Population Scoring

For slide digitization a whole slide imaging (WSI) technique was performed using a digital slide scanner (D-sight 200 fluo, A. Menarini Diagnostics S.r.l. Firenze, Italy). The scanner was calibrated for distance measurements and for background subtraction and white balance. The slides were scanned at 20× magnification and were digitized and stored using the JPEG2000 format.

**Figure 1:**
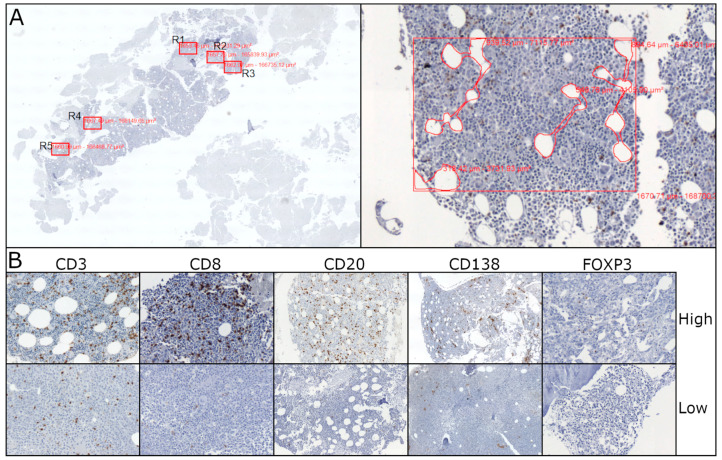
(**A**) The five areas (R1–R5) with the highest densities of CD8+ cytotoxic T cells were selected (2× magnification left, 20× magnification right). Our metric procedure included bone marrow cellularity normalization by deducting bone and fat tissue areas as well as any artifactual empty spaces. The density of each immune cell population was calculated as the mean of the five different areas normalized per mm^2^ and 100% BM cellularity. (**B**) The same areas were chosen for the estimation of CD3, CD20, CD138 and FOXP3. These are some examples that illustrate the variability of the density (high and low) of these immune populations (40× magnification).

In order to ensure the consistency of our metric approach, five areas with the highest densities of CD8+ T cytotoxic (Tc) cells were selected, since these cells represent a well-known anti-neoplastic immune cell population (Figure 1) [10]. The same areas were chosen for the estimation of CD3+, CD20+, CD138+ and FOXP3+ cells. Our metric procedure included BM cellularity normalization by deducting bone and fat tissue areas as well as any artifactual empty spaces. The density of each immune cell population was calculated as the mean of the five different areas normalized per mm^2^ and 100% BM cellularity. The density of CD4+ T helper (Th) cells was estimated by deducting the number of CD8+ cells from the number of CD3+ cells that were counted in serial sections. Moreover, as Foxp3 (+) T regulatory cells (Tregs) seem to play an important role in the immune environment of myelodysplastic BMs [11,12], the number of this cell population was also evaluated in the five regions with the highest densities, irrespective of CD8. Finally, we quantified the lymphoid aggregates, which were defined as clusters (>50 cells) of B and T lymphocytes, some of them with germinal centers.

### 2.4. Statistical Analysis

Statistical analyses were performed with SPSS software (v.25, SPSS Inc., Chicago, IL, USA). Non-parametric statistical tests (Mann–Whitney U test, Kruskal–Wallis) were used to compare differences in scale variables among the groups. The OS and LFS (Kaplan–Meier) for categorical variables were determined and comparisons were made using the log-rank test. For scale variables, the median and the optimal cutoff using x-tile were used to create dichotomous variables for Kaplan–Meier analysis [13]. A univariate Cox regression model was used to identify possible independent prognostic factors for OS and LFS after initiation of 5-AZA treatment. For the multivariate Cox regression models, only significant parameters of the univariate analysis were used. A binary logistic regression analysis was used to identify significant predictors of response to 5-AZA therapy. A *p*-value of <0.05, according to 2-sided tests, indicated significant difference in all cases.

## 3. Results

### 3.1. Lower Densities of Adaptive Immune Cell Subpopulations Characterize the Microenvironment of HR-MDS Compared to Age-Matched Normal Controls

The densities (number of cells/mm^2^) of CD3+ T, CD8+ Tc and CD20+ B cells were significantly lower in the HR-MDS group compared to the control group (*p* = 0.048, *p* = 0.036 and *p* = 0.009, respectively, Table 2). Additionally, the densities of Tregs (Foxp3+) and the ratios of Tregs/T (Foxp3/CD3) and Tregs/Tc (Foxp3/CD8) were also significantly lower in the HR-MDS group (*p* < 0.001, *p* < 0.001 and *p* = 0.001, respectively).

**Figure 2:**
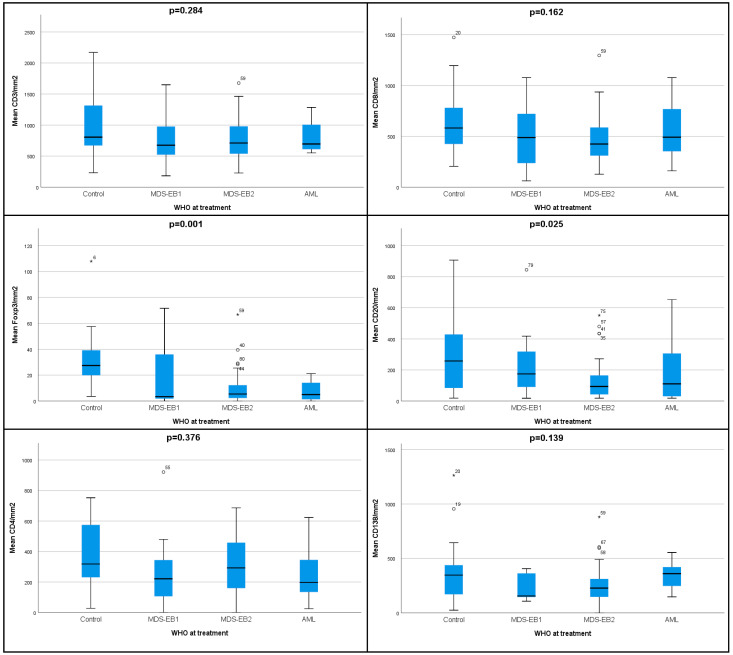
The densities (mean value/mm^2^ of bone marrow) of the immune cell populations according to WHO-defined entities. Individuals from the control group are also included.

There was a significant gradual decrease of Tregs density among the controls, MDS with excess blasts (EB)-1, MDS-EB-2 and AML patients (*p* = 0.001, Figure 2, Supplementary Table S1). Similar decreasing densities were observed for the Tregs/T (%) ratio (*p* = 0.002), as well as the Tregs/Tc (%) ratio (*p* = 0.003). For B cells’ density, we observed again the highest density in the controls, a gradual decrease in MDS-EB-1 and EB-2 (*p* = 0.025) and a slight increase in AML.

### 3.2. Higher Densities of CD3+ T Cells and CD4+ Th Cells Correlate with Response to Treatment with 5-AZA

We categorized the patients according to their treatment response into the following groups: Complete Response/Partial Response/Hematological Improvement (CR/PR/HI), Stable Disease (SD) and Progressive Disease (PD). We identified decreasing densities of CD3+ T cells and Th cells among the groups (*p* = 0.009, Table 3). Furthermore, we observed decreasing ratios of peripheral blood lymphocytes/monocytes (*p* = 0.005) and decreasing values of hemoglobin (Hb) (*p* = 0.010).

When we separated the patients into two groups—non-progressors (CR/PR/HI, SD) and progressors (PD)—the non-progressors displayed higher densities of CD3+ T cells and Th cells (*p* = 0.009 [807 vs. 647] and *p* = 0.009 [313 vs. 212], respectively) and lower ratios of Tregs/T (*p* = 0.039 [1.37 vs. 2.38], Supplementary Table S2). Furthermore, we noted an increased ratio of lymphocytes/monocytes (*p* = 0.023) in peripheral blood and higher numbers of platelets (*p* = 0.023).

### 3.3. Immune Cell Densities Can Aid in Response Prediction

A binary logistic regression model was utilized to identify variables that could be used to predict responders. Responders were more commonly found in the groups with higher CD3+ T (>700 cells/mm^2^, *p* = 0.017) and Th densities (>258 cells/mm^2^, *p* = 0.001), lower Tregs/T ratios (<1.4%, *p* = 0.017), favorable karyotypes (*p* = 0.026), higher lymphocytes/monocytes ratios (*p* = 0.001) and higher baseline Hb values (*p* = 0.027). Using all these parameters in a multiple regression analysis, we identified only the Tregs/T (0.96% in responders vs. 1.83% in non-responders) and lymphocytes/monocytes ratios (24% in responders vs. 9% in non-responders) as significant parameters (*p* = 0.033 and *p* = 0.015, respectively). The model improved the overall prediction accuracy from 66% to 84.6%. Similarly, non-progressors were more common in the groups with higher Tc and Th cell densities (>225 cells/mm^2^, *p* = 0.041 and >241 cells/mm^2^, *p* = 0.015) and increased ratios of lymphocytes/monocytes (>9%, *p* = 0.016) and PLTs (>100.000, *p* = 0.031). When we included all parameters in the model, only Th density was borderline significant (*p* = 0.051), but the model could correctly predict the responses in 83.9% of the patients (75% was the baseline).

### 3.4. Immune Cell Densities Correlate with Overall Survival

Using Kaplan–Meier estimates, we evaluated the impact on OS, dichotomizing patients into two groups using either the population median or the optimal cut-off from X-tile. Patients who had higher densities of T cells (>475 cells/mm^2^ X-tile) and Tc (>225 cells/mm^2^ X-tile) had increased OS (30.2 vs. 7.5 months *p* < 0.001, 31.1 vs. 7.4 months *p* < 0.001, respectively). Similarly, increased OS was noted in patients with a higher density of Th cells, using either the median or the optimal cut-off (> 241 cells/mm^2^, *p* = 0.017, 35.1 vs. 17.4 months and >250 cells/mm^2^ X-tile, *p* = 0.016, 35.7 vs. 17.5 months, respectively). Similar results were obtained for increased CD138+ plasma cell densities (>200 cells/mm^2^, *p* = 0.025, 33.4 vs. 17.8 months, respectively). On the contrary, lower densities of B cells and Tregs correlated with increased OS (<450 cells/mm^2^, X-tile, *p* = 0.035, 29.7 vs. 11.7 months and <11 cells/mm^2^, X-tile, *p* = 0.019, 32.1 vs. 18.1 months, respectively). Increased OS was also identified in patients with a lower ratio of Tregs/T (<4% X-tile, *p* < 0.001, 30.4 vs. 9.1 months). Finally, patients with lower bone marrow cellularity (<90%, X-tile) displayed increased OS (31.1 vs. 9.3 months, *p* = 0.002).

### 3.5. Lower Densities of Tregs and Plasma Cells Correlate with Increased Leukemia-Free Survival

Increased LFS correlated with lower densities of Tregs and plasma cells (<11 cells/mm^2^, X-tile, *p* = 0.022, 44.8 vs. 26.3 months and <360 cells/mm^2^, X-tile, *p* = 0.001, 46.2 vs. 17.8 months, respectively). Similar results were obtained by using the highest Tregs density methodology approach, with a cutoff of 21 cells/mm^2^ (48.5 months vs. 23.1 months, *p* = 0.015). Furthermore, those patients also exhibited lower ratios of Tregs/T cells using either the median (0.78%, *p* = 0.038, 49.4 vs. 28.8 months) or the optimal cut-off (>1%, X-tile, *p* = 0.007, 50.6 vs. 25.3 months) and Tregs/Tc (0.125 median, *p* = 0.035, LFP 46.5 vs. 28.9 months). In addition, patients with lower BM cellularity had increased LFS (<80% median, *p* = 0.014, 49.4 vs. 23.9 months).

### 3.6. Lower Densities of T Cells and Higher Densities of Tregs Correlate with Increased Hazard Ratios of Death and Transformation into AML

Using Cox regression analysis, we identified all important parameters for OS and LFS. Patients had higher risk of death when they displayed BM cellularity >90% (*p* = 0.004, HR: 3.566), increased density of Tregs (>11 cells/mm^2^, *p* = 0.022, HR:2.106) and B cells (>450 cells/mm^2^, *p* = 0.045, HR:2.949). The same was observed when they exhibited a higher ratio of Tregs/T cells than the median or the optimal cut-off (>0.8%, *p* = 0.019, HR: 1.194 and >4% X-tile, *p* = 0.001, HR:4.291, respectively). On the contrary, patients with high T cell density (>475 cells/mm^2^), Th (>250 cells/mm^2^), Tc (>225 cells/mm^2^) and plasma cells (>200 cells/mm^2^) showed a decreased risk of death (*p* < 0.001, HR: 0.195; *p* = 0.018, HR: 0.473; *p* < 0.001, HR: 0.178 and *p* = 0.028, HR: 0.028, respectively). In multivariate Cox regression analysis with IPSS-R and age, the densities of T cells, Th, Tc and Tregs remained significant, whereas ECOG-PS, BM cellularity and B-cells did not (Supplementary Excel S1—Sheet“OS Multi IPSS-R”).

Patients with BM cellularity >80% (population median) had a higher risk of developing AML (*p* = 0.019, HR:3.635). The same was identified for increased densities of Tregs groups dichotomized either with the median (*p* = 0.017, HR: 1.029) or with the optimal cut-off (>11 cells/mm^2^, X-tile, *p* = 0.027, HR:2.666). Similar results were obtained for the ratio (%) of Tregs/T cells (*p* = 0.024, HR: 1.29) and their best cut-off (>1%, X-tile, *p* = 0.011, HR: 3.18). Since we already found that patients who progressed to AML had higher plasma cell densities, we identified the optimal cut-off for LFS (>360 cells/mm^2^, *p* = 0.003, HR: 4.02). In a multivariate Cox regression analysis that included age and IPSS-R, the parameters of Tregs densities, Tregs/T ratio and plasma cell densities remained significant (Supplementary Excel S1—Sheet“LFS Multi IPSS-R”).

### 3.7. An Immune-IPSS-R Score for Enhanced OS and LFS Prediction

We then combined the original IPSS-R score with the data from the immune micro-environment. We tested the IPSS-R score, first as a scale variable and then by dichotomizing patients as very high risk vs. the rest. As expected, both Cox regression and Kaplan–Meier analysis showed significant associations with OS. Then we started adding the significant (in univariate analysis) parameters one by one, using the dichotomous groups with the optimal cut-offs identified in x-tile.

**Figure 3:**
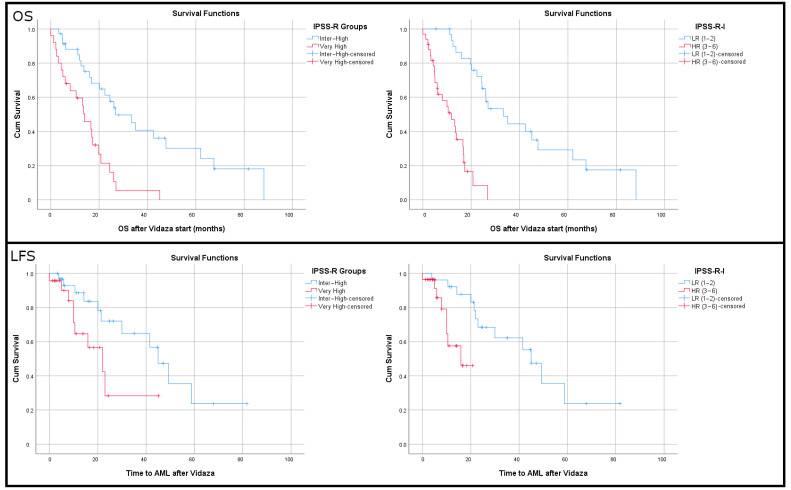
Kaplan–Meier curves showing the overall survival (**upper panel**) and leukemia-free survival (**lower panel**) according to both the IPSS-R groups and the newly proposed IPSS-R-I groups.

Patients with very high or intermediate/high IPSS-R and adverse immune markers were categorized as high risk and those with intermediate/high IPSS-R and favorable immune markers were grouped in the low risk category. After testing several scenarios (Supplementary Excel S1—Sheet\“OS I-IPSS-R”), the best results were obtained when a sum was constructed (Initial IPSS-R [inter or high = 4, very high = 5] + 1 point for every adverse effect [Tregs high, B high]), −1 point for every favorable event [T cell high, Th high, Tc high]. The final model (Figure 3) dichotomized patients into low risk (IPSS-R-I = 1–2) with an OS of 42.41 months (95% CI: 31.78–53.04) and high risk (IPSS-R-I = 3–6) with an OS of 11.18 months (95% CI: 8.37–13.98, *p* < 0.001). The initial IPSS-R of the dichotomous categories showed OS values of 39.36 (95% CI: 28.01–50.71) and 14.6 (95% CI: 10.02–19.25), respectively (*p* = 0.001).

The same score used for LFS outperformed the IPSS-R. Patients grouped by their IPSS-R score had a median LFS of 45.8 months for intermediate–high and 23.1 months for very high (*p* = 0.048). The same patients with IPSS-R-I had a median LFS of 45.8 months vs. 14.7 months (*p* = 0.006). Cox HR was 2.541 (95%CI 0.976–6.646) for very high IPSS-R and 5.168 for high-risk IPSS-R-I patients (95%CI 1.437–18.583).

## 4. Discussion

The main finding of our study is the correlation of immune BM markers with response to treatment and outcome in HR-MDS patients treated with 5-AZA. We further developed a prognostic system, the IPSS-R-I, that incorporates immune markers and increases the efficiency of the IPSS-R.

To the best of our knowledge, only a few recent studies have focused on immunophenotypical characterization of BM trephine biopsies of MDS or AML patients [14,15,16]. On the other hand, there are several studies that use flow cytometry (FC) to measure immune cells in the context of MDS/AML, with many of them using peripheral blood. Using IHC in trephine BM biopsies, rather than FC in BM aspirates, has the advantage of parallel evaluation of BM cellularity, histological architecture, immune cell topography and cell population density per area. Furthermore, hemodilution due to peripheral blood contamination, which occurs to a variable degree in aspirates and may impact the results, is avoided. In addition, it is known that the distribution of immune cell subsets and the expression of immune checkpoint receptors in peripheral blood are not representative of the findings in BM, at least in AML, rendering BM samples more appropriate for immune profiling [17].

We showed that HR-MDS is characterized by low densities of T and Tc cells, B cells and Tregs in BM, a fact that may have an impact on immunosurveillance [18]. The low densities of adaptive immune cells might be related to angiogenesis, which is increased in HR-MDS and is a known immunosuppressive factor in the tumor microenvironment, acting as a barrier to adaptive immune cell infiltration [19,20].

In our study, Tc cells exhibited a gradual decrease according to the original WHO diagnosis, from EB-1 to EB-2 and AML. This tendency was also demonstrated with the same methods in a recent paper by Giovazzino et al., who showed an increased BM recruitment of Tc cells in LR-MDS, the number of which was reduced in HR-MDS [21].

We also demonstrated increased densities of T cells in patients who responded to 5-AZA and had prolonged OS. This finding is in accordance with a recent study by Pescia et al., who also used IHC to evaluate lymphocytic infiltrate in the BM of HR-MDS patients receiving 5-AZA. They also linked the lymphocytic infiltrate with better outcomes and a lower risk of AML progression, proposing a protective role of the reactive infiltrate in regard to leukemic progression [14].

In vitro depletion of CD8+CD57+T cells has been shown to result in colony formation of BM mononuclear cells and, in cases of LR-MDS patients with an abnormal karyotype, in an increase of clonal cells, supporting the inhibitory nature of CD8 Tc [22]. Expansion of this cell population might have therapeutic implications for MDS. This might be related to the recognition of tumor antigens [epitopes like Wilms tumor 1(WT-1), proteinase-3, cancer-testis antigens (CTAs)] by specific anti-tumoral CD8+ T cells that target clonal hematopoietic cells and inhibit disease evolution. Several clinical trials involving antigen vaccination or tumor-specific T-cell adoptive transfer have used the induction of tumor-specific CD8+ T cells to achieve a clinical response. In addition, neoantigen-specific CD8+ T cells, which are proliferated in vitro and able to kill tumor cells, have been infused into MDS patients, with promising results [23,24,25,26]. Taking into consideration the above, HR-MDS patients may be suitable candidates for immunotherapies utilizing CD8+ T cells.

We also showed that HR-MDS is characterized by lower densities of Tregs and lower Tregs/T and Tregs/Tc ratios compared to controls, as well as the gradual decrease in Tregs noted in the WHO classification (from EB-1, EB-2 to AML). Within HR-MDS cases, we found that lower densities of Tregs and lower Tregs/T and Tregs/Tc ratios were associated with better OS and prolonged LFS in 5-AZA-treated patients, whereas increased densities of Tregs and a higher Tregs/T ratio correlated with higher risks of death and AML transformation. Many studies, mainly using flow cytometry, have demonstrated the role of Tregs in MDS pathogenesis. In LR-MDS their number is decreased, contributing to an inflammatory environment that might be related to ineffective hematopoiesis, whereas in HR-MDS increased densities of Tregs may contribute to disease progression by facilitating the immune escape of malignant cells [11,12,27]. Therapeutic interventions such as the use of lenalidomide, which has been shown to reduce Treg numbers, may enhance anti-tumor immune responses in the context of HR-MDS and may enhance the efficacy of the 5-AZA treatment approach. In this way, the densities of Tregs can indicate the immune status of MDS patients and may serve as biomarker for disease progression. Although a disadvantage of our single-color immunohistochemical approach is that all Foxp3+ cells were considered Tregs, we used two different metrics to count foxp3+ cells and both showed the same results.

Additionally, we found a trend of decreased Th cell density in HR-MDS compared to controls. In our cohort of HR-MDS patients, we showed that a higher density of Th cells was associated with a better response to treatment, better OS and a lower risk of death. A previous study of LR and HR-MDS patients found higher Th densities in these patients compared to controls by using flow cytometry, a different experimental approach [28]. Even if the role of this subpopulation of T- cells is underrated in immune neoplastic microenvironments, during the last decade it has been suggested to be crucial in solid tumor pathogenesis [29,30].

Regarding B lymphocytes, their density was inversely correlated with EB status and OS in the 5-AZA-treated patients in our study. Reduced numbers of B cells and their precursors have been demonstrated in LR-MDS [16,31,32], which may indicate a role in the autoimmune disorders observed in MDS patients. Several studies have found a similar association with OS in patients, mainly in solid tumors [33].

In accordance with previous reports using the same methodology [34], no significant differences in the number of lymphoid aggregates were detected between the HR-MDS patients and the control group, and there were no correlations with the clinical parameters. Even though a recent study suggests a potential prognostically poor role, the results do not reach statistical significance [34]. Numerous studies have shown an ambiguous correlation (mostly positive) between tertiary lymphoid structures (their number, location, composition and maturity) and prediction and response to therapy in solid tumors [35].

For the terminal differentiated B cells (plasma cells), little is known about their involvement in MDS pathogenesis. We observed no essential differences in their frequency between the HR-MDS patients and the control group, a fact that was also confirmed in a recent study using multispectral imaging in BM [36]. Of interest is the fact that we correlated the lower density of plasma cells in BM with increases in LFS, as well as with a higher risk of death. Due to a lack of data in the literature, further studies should be performed to verify this result.

The IPSS-R is a standard tool for the prediction of survival in MDS patients. Further enhancements have been suggested using additional clinical and hematological parameters [37]. In our study, by using routine IHC tests and the already established clinicopathological parameters included in the IPSS-R score, we were able to enhance the prediction of responders (CR/PR/HI) and non-progressors (CR/PR/HI/SD) by almost 20% and 10%, respectively.

We showed that the inclusion of immune cell densities could further improve the hazard ratio and OS of grouped patients compared to the IPSS-R. The most significant changes were identified when we constructed a new score (IPSS-R-I) using the densities of Tregs, B, T CD3, CD4 and CD8 cells. The final model, which dichotomized patients into IPSS-R-I low risk and IPSS-R-I high risk, outperformed the initial IPSS-R, having a twice-increased HR for high-risk patients.

Until now, there has been no consensus regarding the integration of immune parameters into the stratification of MDS using either flow cytometry [38] or multi-omics-driven analysis and other molecular techniques [3]. However, in clinical routine the proposed IPSS-R-I relies only on immunohistochemistry and could act as a practical tool for better prognostication of 5-AZA-treated HR MDS patients. We are aware of the limitations of this study, realizing that this is not a complete summary of the entire immune biology and accepting that several other tumor microenvironmental parameters, such as myeloid-derived suppressor cells, mesenchymal stem cells, monocytes/macrophages and dendritic cells, play important roles in MDS pathogenesis. Furthermore, the determination of robust cut-off values for patient stratification requires a significant number of cases with user-independent methods such as whole slide scanning and computational pathology.

## 5. Conclusions

Immunohistochemical BM immune profiling represents a powerful and easily useable tool for investigating the possible role of bone marrow immune microenvironment in the pathogenesis and progression of MDS, but also its association with clinical outcome. Additionally, potent immunotherapeutic strategies such as bispecific antibodies, immune checkpoint inhibitors and autologous chimeric antigen receptor therapies may also rely on a preserved immune cell population in order to achieve a response to treatment [39].

## Figures and Tables

**Table 1:** Patients’ clinical characteristics.

Patient Characteristics	Groups	No.	Percent	Median (Min–Max)
Sex	Male	51	60.7%	
	Female	33	39.3%	
Age (N = 84)				73 (43–90)
Age groups	≤70	30	35.7%	
	>71	54	64.3%	
WHO-defined entities	EB1	12	18.75%	
	EB2	44	68.75%	
	AML	8	12.50%	
Performance status (ECOG)	0–1	38	84.4%	
	2+	7	15.6%	
Karyotype risk groups	Good	40	61.5%	
	Intermediate	8	12.3%	
	Poor	17	26.2%	
Neutrophils (10^9^/Lt) (N = 64)				0.95 (0.1–17.76)
Monocytes (10^9^/Lt) (N = 63)				0.17 (0–11.23)
Lymphocytes (10^9^/Lt) (N = 63)				1.29 (0.11–5.13)
Lymphocyte (10^9^/Lt) groups	<1.0	18	28.6%	
	>1.0	45	71.4%	
Ratio lymphocytes/monocytes (N = 61)				7.64 (0.32–157)
Ratio lymphocytes /neutrophils (N = 62)				1.15 (0.13–7.6)
Ratio lymphocytes/neutrophils (X-tile)	<0.4	9	14.5%	
	>0.4	53	85.5%	
Platelets (N = 61)				70 (8–534)
Feritin (mg/mL) baseline (N = 53)				440 (34–4255)
Hb (g/dL) (N = 64)				8.85 (5.9–11.8)
Hb (g/dL) groups	>10	13	20.3%	
	8–10	37	57.8%	
	<8	14	21.9%	
No. of cytopenias (N = 62)				2 (0–3)
BM blasts (%) (N = 63)				11 (1–50)
IPSS-R score (N = 60)				6.0 (4–9.5)
IPSS-R groups	Intermediate	3	5.0%	
	High	32	53.3%	
	Very High	25	41.7%	
LDH (N = 65)				280 (129–1267)
LDH groups	Normal	21	32.3%	
	Abnormal	44	67.7%	
Overall response groups (N = 65)	CR/PR/HI	20	30.8%	
	SD	29	44.6%	
	PD	16	24.6%	
Time of best response (months) (N = 18)				6 (1–12)
Total cycles of Aza given (N = 57)				8 (1–49)
Transformation into AML (N = 55)	No	34	61.8%	
	Yes	21	38.2%	
Time to AML after Vidaza (N = 55)				14.2 (0–81.7)
Status (N = 65)	Alive	18	27.7%	
	Dead	47	72.3%	
Follow-up OS after Vidaza start (months) (N = 64)				16.59 (0–88.16)
Follow-up LFS after Vidaza start (months) (N = 54)				14.07 (0–81.70)

**Table 2:** Immune cell densities in patients and controls.

Parameters	Patients	Controls	*p* (Mann–Whitney U)
CD3 (mean/mm^2^)	764	977	0.048
CD4 (mean/mm^2^)	286	376	0.143
CD8 (mean/mm^2^)	477	640	0.036
Foxp3 (mean/mm^2^)	11.5	32.4	0.000
CD20 (mean/mm^2^)	161	308	0.009
FoxP3/CD3 (%)	1.64%	3.42%	0.000
FoxP3/CD8	0.025	0.052	0.001

**Table 3:** Parameters that predict response to 5-Azacytidine treatment.

Parameter	CR/PR/HI	SD	PD	*p* (Kruskal–Wallis)
CD3 (mean/mm^2^)	852	776	647	0.018
CD4 (mean/mm^2^)	344	292	212	0.020
Lymphocytes/monocytes	24	10.6	5.8	0.005
Hb (g/dL)	9.3	8.9	8.4	0.010
Time to AML (months)	34.8	19.2	8.3	0.012
OS (months)	36.6	16.1	11.6	0.001

## Data Availability

The dataset is available on request from the authors.

## Supplementary Materials

The following supporting information can be downloaded at: https://www.mdpi.com/article/10.3390/cancers17071104/s1, Table S1: Differences among WHO groups Table S2: Differences among progressors and non-progressors; Excel S1: Statistical analysis.
