# Supplementary material for: Prognostic Role of Adaptive Immune Microenvironment in Patients with High-Risk Myelodysplastic Syndromes Treated with 5-Azacytidine
Source: Cancers (Basel). 2025 Mar 25;17(7):1104. doi: 10.3390/cancers17071104 (PMC11988087; doi:10.3390/cancers17071104)
Supplement: Supplementary file 1 [file cancers-17-01104-s001.zip › Supplementary Tables S1 and S2.pdf]

Supplementary Table S1. Differences among WHO grouping

Variables at diagnosis	Controls	MDS-EB1	MDS-EB2	AML	p (Kruskal-Wallis)
Cellularity histology report %	37.8	70.7	76.7	72.5	0.001
Blasts histology report %	1.2	6.6	10.9	20.3	0.001
Lymphoid aggregates/cm	0.35	0.98	0.70	0.15	0.067
Mean CD3/mm^2	977.5	763.2	761.1	807.8	0.284
Mean CD8/mm^2	640.1	502.1	457.9	557.5	0.162
Mean CD4/mm^2	376.4	271.8	303.6	250.3	0.376
Mean Foxp3/mm^2	32.4	18.9	9.8	7.7	0.001
Mean CD20/mm^2	308.8	241.7	135.3	195.7	0.025
Mean CD138/mm^2	379.3	237.0	258.5	345.1	0.139
FoxP3/CD3 (%)	3.42%	2.49%	1.45%	0.83%	0.002
CD8/CD3 (%)	65.64%	64.11%	63.24%	69.38%	0.810
FoxP3/CD8	0.052	0.036	0.024	0.016	0.003

High
Intermediate
Intrer-Low
Low

Supplementary Table S2. Differences among progressors and non-progressors.

Null Hypothesis	Non-progressors	Progressors	p (Mann-Whitney U Test)
Mean CD3/mm^2	807	647	0.009
Mean CD4/mm^2	313	212	0.009
Mean FoxP3/CD3 (%)	1.37	2.38	0.039
Hb (g/dl)	9.1	8.4	0.034
Ratio Lymphs/Mono	164	5.8	0.023
PLTs	129	84	0.033
Hb (g/dl) Baseline	9.6	8.7	0.047
